# Sortase-mediated chemical protein synthesis reveals the bidentate binding of bisphosphorylated p62 with K63 diubiquitin†
†Electronic supplementary information (ESI) available. See DOI: 10.1039/c7sc02937c



**DOI:** 10.1039/c7sc02937c

**Published:** 2017-08-04

**Authors:** Xiang-Long Tan, Man Pan, Yong Zheng, Shuai Gao, Lu-Jun Liang, Yi-Ming Li

**Affiliations:** a Tsinghua-Peking Center for Life Sciences , Ministry of Education Key Laboratory of Bioorganic Phosphorus Chemistry and Chemical Biology , Department of Chemistry , Tsinghua University , Beijing 100084 , China; b School of Biological and Medical Engineering , Hefei University of Technology , Hefei , Anhui 230009 , China . Email: ymli@hfut.edu.cn; c High Magnetic Field Laboratory , Chinese Academy of Sciences , Hefei 230031 , China

## Abstract

Phosphorylation of S403 or S407 of the autophagic receptor protein p62 has recently been discovered to enhance the binding of p62 with ubiquitinated protein substrates to upregulate selective autophagy. To elucidate the molecular mechanism of how phosphorylation regulates the recruitment of ubiquitinated proteins, we report the first chemical synthesis of homogeneously phosphorylated p62, which enables the setting up of accurate *in vitro* systems for biochemical studies. Our synthesis employs the technology of sortase A-mediated protein hydrazide ligation, which successfully affords three types of phosphorylated p62 at the multi-milligram scale. Quantitative biochemical measurements show that the binding affinity of S403/S407-bisphosphorylated p62 to K63 diubiquitin is significantly higher than that of mono-phosphorylated p62. This finding suggests that phosphorylated S403 and S407 sites should bind to different epitopes on the ubiquitin chain. Furthermore, glutamate mutation is found to give a significantly impaired binding affinity, implying the necessity of using chemically synthesized phosphorylated p62 for the biochemical study of selective autophagy.

## Abstract 2

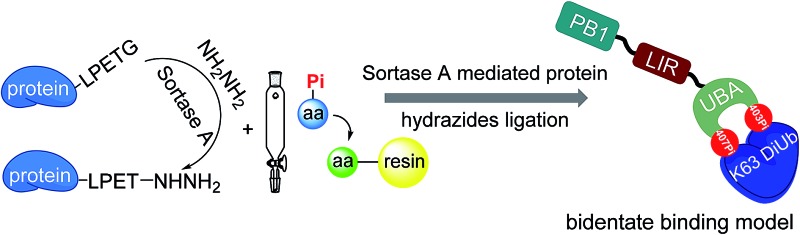
This work reports the first chemical synthesis of the phosphorylated p62 protein and reveals a bidentate binding model of bisphosphorylated p62.

## Introduction

As a conservative process of degradation in eukaryotes, selective autophagy regulates a series of physiological processes such as the renewal of damaged organelles, the degradation of protein aggregates, and the removal of exogenous microorganisms.^1^ In selective autophagy, the substrate in the cytoplasm is selectively captured into an autophagosome with a bilateral membrane structure and subsequently delivered to the lysosome for degradation.^2^ The specific identification of autophagic substrates during this process is derived from autophagy receptor proteins such as p62/SQSTM1 (sequestosome-1).^3^ In the process, p62 works as a tethering factor that can recognize ubiquitinated autophagic protein substrates and present them into the autophagosome through binding to the microtubule-associated protein 1 light chain 3 (LC3), enabling selective degradation of the substrates.^3*a*^ In 2011, Matsumoto *et al.* found that phosphorylation of serine (Ser) 403 of p62 can increase its binding affinity to the K63 ubiquitin (Ub) chain, resulting in up-regulation of autophagy levels.^4^ More recently, p62 phosphorylation at Ser407 was also reported by Yue’s group, which again can increase the ability of p62 to bind K63 ubiquitin and upregulate the levels of autophagy.^5^ Yue *et al.* further revealed that the S403 and S407 sites can both be phosphorylated, thereby breaking the dimerization of the UBA domain to promote its S403 site phosphorylation.^5^

The above findings raise a number of interesting questions. Firstly, what role does the phosphorylation of each serine play in the recruitment of ubiquitinated substrates? Secondly, will bisphosphorylation bring about a different substrate recruitment effect? Finally, what is the molecular mechanism for the enhanced binding of the phosphorylated p62 to the ubiquitin chain; or more specifically, does phosphorylated S403 or S407 bind to the same epitope on the ubiquitin chain? Answers to these questions are of critical value for a more accurate understanding of the role of phosphorylation in selective autophagy, and the knowledge may provide a framework for the development of biochemical tools to modulate selective autophagy. A bottleneck for studying these problems lies in the lack of *in vitro* biochemical systems to investigate the molecular recognition of phosphorylated p62 by the K63 ubiquitin chain. This demands the acquisition of different types of phosphorylated p62.

Chemical protein synthesis provides a valuable tool for obtaining homogeneous proteins, especially proteins with post-translational modifications (PTMs).^6^ For instance, Brik *et al.* successfully prepared the Tyr57-phosphorylation of histone H2A by chemical total synthesis.^7^ Payne *et al.* described the generation of sulfated chemokine binding proteins through an efficient one-pot total synthesis.^8^ In addition to total synthesis, the Schwarzer and Pentelute groups have developed a sortase-mediated protein semi-synthesis strategy for acquiring homogeneous histone H3 and lethal factors.^9^ These studies demonstrate that chemical protein synthesis is an effective way to incorporate PTMs at any desired position in the target protein.

In the present work, we report the first chemical synthesis of three types of phosphorylated p62 protein at the multi-milligram scale. The synthesis was achieved using the technology of sortase A-mediated protein hydrazide ligation.^10^ Surface plasmon resonance (SPR) was used to quantify the binding affinity between phosphorylated p62 and K63 diubiquitin (diUb). The results indicated that phosphorylation of S403 (p62_S403Pi_) or S407 (p62_S407Pi_) can increase the binding affinity of p62 to K63 diUb from 167.9 ± 13.0 μM to 4.4 ± 0.4 μM or 14.8 ± 1.0 μM by about 34 or 11 fold. More importantly, the binding affinity of bisphosphorylated p62 (p62_2Pi_) to K63 diUb was further increased to 704 ± 63 nM, which represents a 240-fold enhancement compared to un-phosphorylated p62. These findings are mechanistically important, as they may suggest that the phosphorylated S403 and S407 positions of p62 should bind to the ubiquitin chain at different surface epitopes.

## Results and discussion

**Scheme 1:**
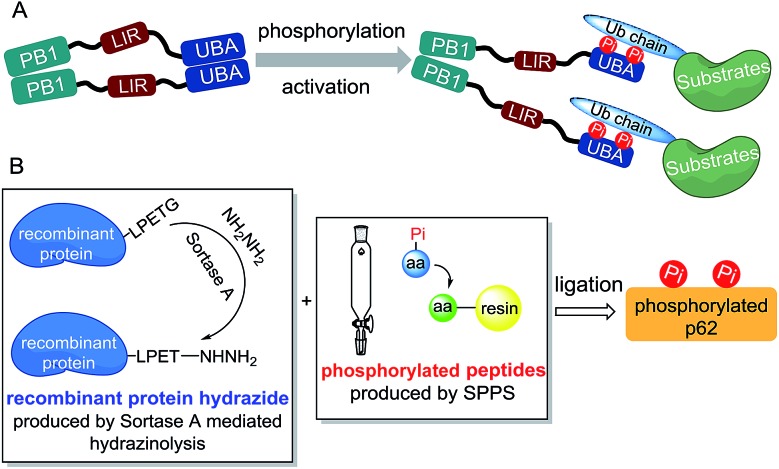
(A) The autophagy-related domains of p62 mainly include PB1, LIR and UBA. During selective autophagy, multiple serine sites of UBA are phosphorylated to break the dimerization of UBA and enhance its binding to ubiquitinated substrates. (B) Phosphorylated p62 can be efficiently obtained through a sortase A-mediated protein chemical semi-synthesis strategy.

Considering the preparation of phosphorylated p62, we first analysed the structural motif of this protein. The autophagy-related domains of p62 mainly include the PB1 domain, the LC3-interacting region (LIR) and the ubiquitin-associated (UBA) domain (Scheme 1A).^11^ Among them, the PB1 domain is responsible for the oligomerization of p62, whereas the LIR motif is responsible for identifying and binding the autophagy-associated protein LC3.^12^ The interaction between p62 and the autophagic substrates during ubiquitination is mediated by the UBA domain.^13^ To avoid the interference caused by p62 oligomerization, we chose a truncated body (amino acids 320–436) with the LIR motif and the UBA domain as the synthetic target.

Although *in vitro* enzymology is an effective method to obtain phosphorylated proteins, the site selectivities of specific kinases (CK2, ULK1 and TBK1) for the UBA domain phosphorylation of p62 are not high, making it difficult to obtain homogeneously phosphorylated p62.^4^ Other techniques such as amber codon suppression can also be used to obtain phosphorylated proteins, but embedding of a modified stop codon in cDNA lowers the expression level and may not be effective for obtaining proteins with double phosphorylation.^14^ Thus we decided to obtain p62_S403Pi_ and p62_S407Pi_ through chemical protein synthesis. Initially we tried total chemical synthesis, but we were not able to make the N-terminal peptide (amino acid 326–353) using either conventional stepwise standard 9-fluorenylmethoxycarbonyl-based solid phase peptide synthesis (Fmoc-SPPS) or microwave assisted synthesis (Fig. S1†). The reason for the difficulty remains unclear, as the N-terminal peptide (amino acid 326–353) does not contain difficult sequences.

**Fig. 1:**
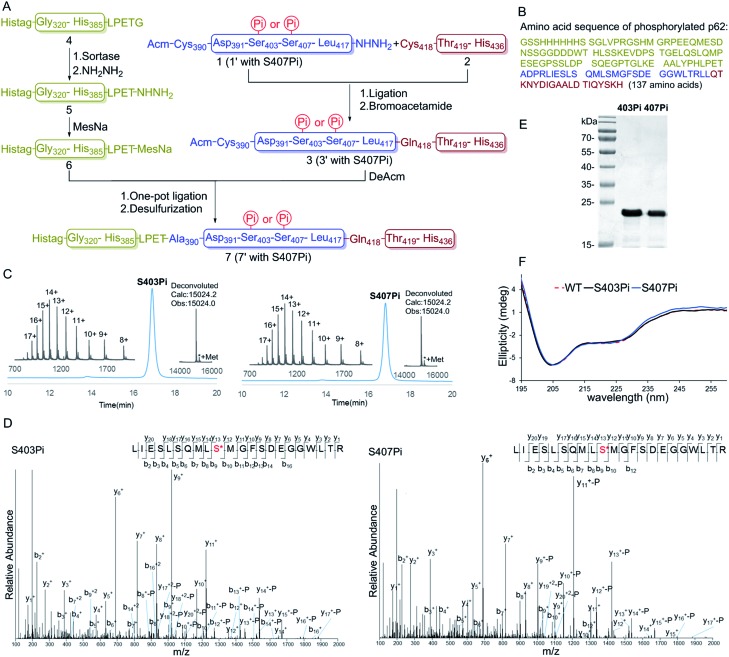
Chemical synthesis of phosphorylated p62 with different phosphorylation sites. (A) General synthetic route. (B) Amino acid sequence of the synthetic phosphorylated p62. (C) Analytical HPLC chromatograms (*λ* = 214 nm) and ESI-MS spectra of the isolated products **7** and **7′**. (D) ESI-MS/MS spectra of the products **7** and **7′**. (E) SDS-PAGE analysis of p62_S403Pi_ and p62_S407Pi_. (F) CD spectra of p62_S403Pi_ and p62_S407Pi_ compared with p62_WT_.

To overcome the problem, we turned to protein semi-synthesis to circumvent the hard-to-make peptide segment through recombinant expression. We first tested canonical intein-based expressed protein ligation technology.^15^ However, the expression level of the intein fused p62 (G320-E389) segment was very low and we had to abandon this route after a number of failed attempts. At this point we turned to the idea of using sortase-mediated protein ligation (SML), which has been demonstrated to be practical in a number of recent studies.^9,10,16^ Traditional SML can be used to modify proteins bearing a short recognition sequence (usually LPXTG). The active-site Cys of sortase cleaves between LPXT and G to produce a thioester intermediate, which reacts with a nucleophile containing one to five Gly residues to afford the ligation product. Recently, our group developed a new version of the sortase-mediated hydrazinolysis reaction of proteins, which can be used to prepare protein hydrazides with high yields by using hydrazine as the nucleophile.^10^ Because the protein hydrazide is no longer a substrate of the transpeptidase, sortase-mediated hydrazinolysis is irreversible and exhibits less hydrolysis. As the sequence (LPPEA) before the phosphorylation site of p62 is similar to the sortase A cleavage site (LPETG), we decided to obtain p62 (G320-H385)-LPET-NHNH_2_ (Fig. 1A, segment **5**) containing two mutation sites (*i.e.* Pro–Glu to Glu–Thr) by using the sortase-mediated hydrazinolysis method (Scheme 1B). Since the two mutated sites are in the disordered region of the protein, we believed that these mutations will not affect the structure and function of p62. The rest of p62, containing two phosphorylation sites, will be divided into two segments (**1** or **1′** and **2** or **2′**) and obtained by total chemical synthesis. Finally, the full-length p62 can be assembled by sequential hydrazide-based native chemical ligation (Fig. 1A).^17^

Segments **1** (or **1′**) and **2** (or **2′**) were obtained by standard Fmoc-SPPS at room temperature, where Glu355 was temporarily mutated to cysteine to facilitate ligation. The ligation between segments **1** (or **1′**, 1.0 eq.) and **2** (1.1 eq.) was performed in the presence of 4-mercaptophenylacetic acid (MPAA, 40 eq.) at pH 6.3 in 6 M Gn·HCl and completed overnight. 2-Bromoacetamide was then added to the reaction mixture for the conversion of the Cys residue at the ligation site to ψ-Gln.^18^ The reaction product was purified by RP-HPLC to afford segment **3** (or **3′**). To obtain segment **5** using the sortase A-mediated protein hydrazinolysis reaction, segment **4** containing the mutated sequence LPETG at the C-terminus was obtained by recombinant expression. Segment **5** was then obtained by adding NH_2_NH_2_ (pH 7.4, 200 mM) and sortase A to produce **5** at a final concentration of 10 μM before it was incubated at 37 °C for 6 hours.^10^ Segment **5** was subsequently converted to thioester **6** in the presence of MesNa (100 eq.). The acetamidomethyl (Acm) group of segment **3** (or **3′**) was removed by PdCl_2_ (15 eq.) in 6 M Gn·HCl at pH 6.8 at 37 °C for 1 hour before the product was ligated with segment **6** in a one-pot fashion.^19^ After final desulfurization in the presence of TCEP (500 mM), tBuSH (equimolar equivalents to sulfur) and 2,2′-azobis[2-(2-imidazolin-2-yl)propane]dihydrochloride (VA-044) as an initiator, the final product **7** (or **7′**) was obtained in 12% overall yield (Fig. 1A).^20^ The purity of the phosphorylated p62 and the correctness of the phosphorylation site were identified by LC-MS, LC-MS/MS and SDS-PAGE respectively (Fig. 1C–E). After refolding by gradient dialysis, the circular dichroism (CD) spectrum showed that the phosphorylated proteins had double negative peaks in the 200–230 nm region, which was almost identical to the recombinant Wide Type (WT) sample, indicating correct folding (Fig. 1F). The well folded phosphorylated p62 could easily be obtained in 20 milligram levels by gel filtration chromatography. Notably, the synthesis of phosphorylated p62 represents the first example of the preparation of PTM proteins using sortase A-mediated protein hydrazide ligation. It also extends the application of SML in protein chemical synthesis, demonstrating that this strategy provides an effective method to obtain target proteins that are difficult to access either by protein total synthesis or intein based semi-synthesis.

**Fig. 2:**
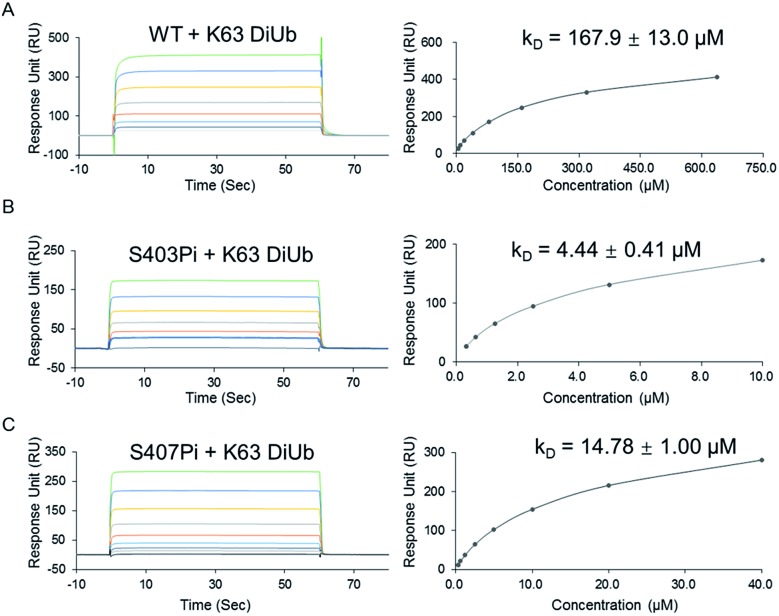
SPR analysis of the monophosphorylated p62 with K63 DiUb. (A) SPR binding studies of p62 WT to K63 DiUb. (B) SPR binding studies of p62_S403Pi_ to K63 DiUb. (C) SPR binding studies of p62_S407Pi_ to K63 DiUb.

With two monophosphorylated p62 proteins in hand, we quantified the binding affinity between modified p62 and K63 diUb. Previous immunoprecipitation tests showed that both p62_S403Pi_ and p62_S407Pi_ could enhance the binding affinity between p62 and K63 diUb.^4,5^ Herein, we measured the binding affinities using SPR, where p62 WT, p62_S403Pi_ and p62_S407Pi_ were immobilized onto CM5 sensor chips by amine coupling. Subsequently, K63 diUb, which was obtained enzymatically,^21^ was passed through the surface of the chip at different concentrations. The results showed that the binding affinity of K63 diUb to p62_S403Pi_ was 4.4 ± 0.4 μM, whereas the binding affinity to p62_S407Pi_ was 14.8 ± 1.0 μM, representing a 34 or 11-fold enhancement over p62 WT (167.9 ± 13.0 μM) (Fig. 2). Thus the phosphorylation at each site exerts a similar enhancement on the binding of p62 with diUb.

**Fig. 3:**
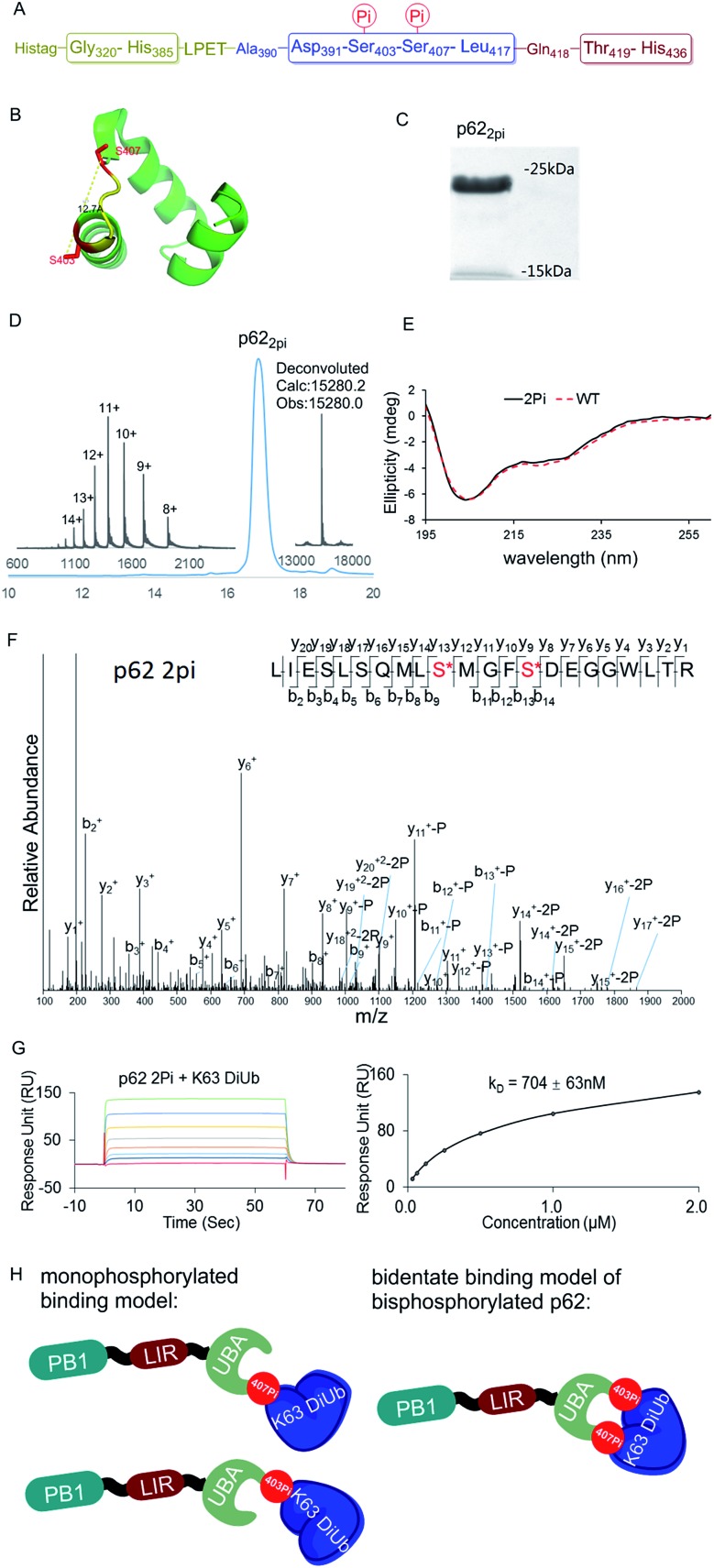
(A) The sequence of p62 with the two phosphorylation sites. (B) The crystal structure of the UBA domain shows that the phosphorylations at S403 and S407 are close to each other. S403 and S407 sites are labeled in red and the MGF flexible sequence is labeled in yellow (PDB: 3b0f). (C) SDS-PAGE analysis of p62_2pi_. (D) Analytical HPLC chromatogram (*λ* = 214 nm) and ESI-MS of isolated p62_2pi_. (E) CD spectrum of p62_2pi_ compared with that of p62_WT_. (F) ESI-MS/MS spectrum of p62_2pi_. (G) SPR binding studies of p62_2Pi_ to K63 diUb. (H) A bidentate binding model of bisphosphorylated p62 binding with K63 diUb.

The above observations prompted us to question whether the two phosphorylation sites of p62 act on the same epitope of ubiquitin. According to the crystal structure of the UBA domain of p62, S403 and S407 are spatially separated only by a short and flexible MGF sequence (Fig. 3B).^22^ If the two phosphorylation sites of p62 act on the same epitope of ubiquitin, they are functionally redundant. To answer the question, we need to obtain bisphosphorylated p62. In this context, we synthesized S403/S407 bisphosphorylated p62 in 9% yield also using sortase-mediated peptide hydrazide ligation (Fig. 3A). LC-MS, SDS-PAGE and LC-MS/MS analysis confirmed the purity of the p62_2pi_ and that the two phosphorylation sites were correct (Fig. 3C, D and F). After refolding, the CD spectrum indicated that p62_2pi_ formed a secondary structure similar to p62 WT (Fig. 3E). We then measured the binding affinity of p62_2pi_ to K63 diUb as 704 ± 63 nM using SPR (Fig. 3G). We were surprised to find that the binding affinity of bisphosphorylated p62 increased by 240 times compared to the 34 or 11-fold increase for p62_S403Pi_ and p62_S407Pi_. This finding suggested that bisphosphorylation of p62 exerted a completely different ubiquitin recruitment effect in selective autophagy compared to monophosphorylation, which may provide another dimension of regulation to control selective autophagy in an orderly manner.^5^ In addition, the enhanced binding also suggested that the two phosphorylated serines should not bind to the same position on the ubiquitin, but instead bind to different epitopes, and thus exert a synergistic function. Therefore, the molecular recognition of p62 with bisphosphorylation can be defined as a bidentate binding mechanism (Fig. 3H).^23^ One typical previous example of the bidentate effect is that deubiquitinase hOtu1 specifically recognizes K48 diUb through binding to both the proximal and the distal Ub of K48 diUb at different binding sites simultaneously.^24^

**Fig. 4:**
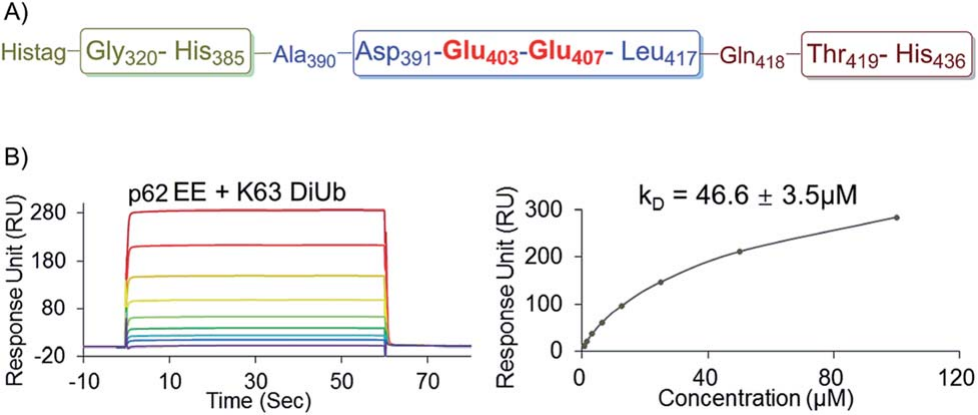
(A) The sequence of the recombinantly expressed p62 with double E mutation. (B) SPR binding studies of p62_EE_ to K63 diUb.

To further elucidate the bidentate binding models of p62_2pi_ with K63 diUb, we need to solve the structure of the protein complex, which will be reported in a follow-up study. At present we want to emphasize that the preparation of phosphorylated proteins is a prerequisite for subsequent structural studies. The reason is that we found that using glutamine (E) mutation instead of natural phosphorylation was unsuitable for modulating the binding between p62 and K63 diUb. Specifically, SPR experiments showed that the binding affinity of the S403E and S407E double mutant of p62 (p62_EE_) to the ubiquitin chain was only 46.6 ± 3.5 μM, while p62_2pi_ gave 704 ± 63 nM (Fig. 3G and 4B). The results indicate that the E mutant mimics will greatly weaken the binding to the ubiquitin chain. This great difference in binding affinity suggests that p62_EE_ is an inappropriate molecular tool either for the construction of p62 and ubiquitin complexes or for the analysis of the contribution of different phosphorylations. Recently, Komander *et al.* also revealed that glutamine mutation of Ub may not reconcile all the effects of Ub Ser65 phosphorylation during phosphorylation in ubiquitin-mediated mitochondrial autophagy.^25^ These observations suggest that the development of chemical protein synthesis methods to obtain natural phosphorylated proteins for the study of autophagy-related protein–protein interactions is an irreplaceable approach.

## Conclusions

In summary, we have described the efficient semi-synthesis of mono and bisphosphorylated p62 proteins through sortase A-mediated protein hydrazide ligation to give the desired product in multi-milligram levels. By taking advantage of our ability to prepare each homogeneous phosphorylated p62, we built the first accurate *in vitro* biochemical system to quantify the binding affinity between phosphorylated p62 and K63 diUb. Quantitative binding experiments showed that compared with p62 WT, p62_S403Pi_ or p62_S407Pi_ can increase the binding affinity by about 34 or 11 fold. Moreover, the binding affinity of p62_2Pi_ to K63 diUb was further enhanced by 240 times to 704 ± 63 nM. The results reveal a bidentate binding model of the p62 protein in the selective autophagy process in which the S403 and S407 sites of bisphosphorylated p62 bind to different epitopes on the ubiquitin chain. It is worth emphasizing that the double-glutamate mutation of p62 significantly reduces the binding affinity with K63 diUb in selective autophagy compared to natural phosphorylation. Our study highlights the importance as well as the power of protein chemical synthesis in enhancing our understanding of autophagy-related protein regulation with multiple PTMs.
