# Supplementary material for: Sortase-mediated chemical protein synthesis reveals the bidentate binding of bisphosphorylated p62 with K63 diubiquitin
Source: Chem Sci. 2017 Aug 4;8(10):6881–7. doi: 10.1039/c7sc02937c (PMC5636944; doi:10.1039/c7sc02937c)
Supplement: Supplementary file 1 [file SC-008-C7SC02937C-s001.pdf]

Electronic Supplementary Material (ESI) for Chemical Science.
This journal is © The Royal Society of Chemistry 2017

Supplementary Information

Sortase-mediated chemical protein synthesis reveals bidentate binding of bisphosphorylated p62 with K63 diubiquitin

Xiang-Long Tan,[a,b] Man Pan,[a] Yong Zheng,[b,c] Shuai Gao,[a] Lu-Jun Liang,[a] Yi-Ming Li [b*]

[a] *Tsinghua-Peking Center for Life Sciences, Ministry of Education Key Laboratory of Bioorganic Phosphorus Chemistry and Chemical Biology, Department of Chemistry, Tsinghua University, Beijing 100084, China;*

[b] *School of Biological and Medical Engineering, Hefei University of Technology, Hefei, Anhui 230009, China, E-mail: ymli@hfut.edu.cn*

[c] *High Magnetic Field Laboratory, Chinese Academy of Sciences, Hefei 230031, China.*

Table of Contents

1. General Information……………………………………………………………….…… S2

a. Materials

b. HPLC and FPLC

c. Molecular biology and biochemistry

d. Mass spectrometry

2. Experimental Section……………………………………………………………….……. S3

a. Peptide synthesis

b. Cloning and protein expression

c. General protocol for protein hydrazide ligation.

d. General protocol for Sortase A mediated peptide hydrazine expression.

e. Protein folding and purification.

f. Circular diagram determination

g. Surface plasmon resonance determination.

h MS/MS identification of specific phosphorylation site.

3. Experimental figures……………………………………………………………….……. S6

1. General Information

A. Materials

Rink amide Resin and 2-Chlorotrityl Chloride Resin were purchased from NanKai Hecheng (Tianjin, China) Ltd. Fmoc-Ser(HPO_3Bzl)-OH as well as 1-Hydroxy-7-azabenzotriazole (HOAt) and other Fmoc-amino acids were purchased from CSBio Co. (Shanghai, China). 1-[Bis(dimethylamino)methylene]-1H-1,2,3-triazolo[4,5-b] pyridinium 3-oxid hexafluorophosphate (HATU), ethyl cyanoglyoxylate-2-oxime (oxyma) were purchased from Aladdin (Shanghai, China). N, N-Diisopropylethylamine (DIEA), N, N-diisopropylethylamine (DIPEA), N, N-Diisopropyl-carbodiimide (DIC), 4-mercaptophenylacetic acid (MPAA) and triisopropylsilane (TIPS) were purchased from Ouhe Technology (Beijing, China). HPLC grade acetonitrile was purchased from J. T. Baker (USA). Guanidine hydrochloride (Gn-HCl), N,N-Dimethylformamide (DMF), HPLC grade trifluoroacetic acid (TFA) were purchased from J&K (Beijing, China). CH_2Cl_2 (DCM), $NaNO_2$ and NaCl were purchased from Beijing Chemical Industry (Beijing, China).

B. HPLC, Mass spectrometry and FPLC

Analytical reverse phase HPLC (SHIMADZU, Prominence LC 20-AT) was used to monitor the purity of peptides and reaction progress with analytical columns (Welch XB C4 and Grace Vydac C8) with flow rate 1.0 mL/min. Semi-preparative RP-HPLC (SHIMADZU) was used to purify crude peptides and reaction products with semi preparative columns (Grace Vydac C4 and welch XB C18) with a flow rate of 6 mL/min and 10 mL/min, respectively.

Peptides and proteins were characterized by standard LC/MS 2020 (SHIMADZU) ESI mass spectrometry. Final products were characterized by high-resolution ESI mass spectrometry on Agilent Time of Flight Mass Spectrometer.

Proteins were purified by AKTA Pure (GE Healthcare) instrument with Superdex 75 or Superdex 200 (GE Healthcare) for gel filtration or Mono S and Mono Q (GE Healthcare) for ion exchange chromatography.

C. Molecular biology and biochemistry

All primers for cloning were purchased from Ruibo Biotech (Beijing, China). Enzymes for cloning were purchased from New England Biolabs (England). LB medium was purchased from Baoruyi Biotech (Beijing, China).

D. Mass Spectrometry

High-resolution ESI mass spectra was measured by the Afilent 6210 Time of Flight Mass Spectrometer. Normal ESI mass spectra was measured by Bruker Daltonics Data Analysis 3.0.

2. Experimental Section

A. Peptide synthesis.

Peptide fragments	Peptide sequence
Acm-Cys_{390}-Ser_{403Pi}-Leu_{417} (**1**)	C(Acm)DPRLIESLSQMLS(Pi)MGFSDEGGWLTRLL
Acm-Cys_{390}-Ser_{407Pi}-Leu_{417} (**1'**)	C(Acm)DPRLIESLSQMLSMGFS(Pi)DEGGWLTRLL
Acm-Cys_{390}-Ser_{403Pi}-Ser_{407Pi}-Leu_{417} (**1''**)	C(Acm)DPRLIESLSQMLS(Pi)MGFS(Pi)DEGGWLTRL L
Cys_{418}-His_{436} (2)	CTKNYDIGAALDTIQYSKH
Gln_{326}-Leu_{353}	QMESDNCSGGDDDWTHLSSKEVDPSTGEL

Table S1 synthetic peptide fragments.

For the preparation of Fmoc-$NHNH_2$, 2-Chlorotrityl Chloride Resin, 8 eq amount of DIPEA and 4 eq amount of Fmoc-$NHNH_2$ were added into 2-Chlorotrityl Chloride Resin which was properly stirred in DCM solution. After 8 hours of stirring and washing with DCM, Fmoc-$NHNH_2$, 2-Chlorotrityl Chloride Resin was obtained with estimated loading of 0.38 mmol/g.

The synthesis of Fragment **1**, **1'** and **1''** were conducted on Fmoc-$NHNH_2$, 2-Chlorotrityl Chloride Resin which were coupled with amino acid (4 eq.), coupling reagent (3.8 eq. HATU), and HOAT (3.8 eq.) in 0.3 M DIEA solution in room temperature. Fmoc-Ser(HPO3Bzl)-OH was coupled to the resin double times with the same strategy. The synthesis of Fragment 2 was prepared on rink amide resin with 0.5 mmol/g substitution which was coupled with amino acid (4 eq.), coupling reagent (Oxyma 4eq. & DIC 4eq.) by using CEM microwave solid phase peptide synthesizer.

After the assembly completed, peptides were cleaved from resin by TFA cocktail (88% TFA, 5% H_2O, 5% Phenol, 3% EDT, 5% Phenylsulfide), and then analyzed and purified by reversed-phase HPLC (CH_3CN-H_2O, 0.1% TFA), molecular weight of each peptide was

confirmed by electrospray ionization (ESI) mass spectrometry (MS).

B. Cloning and Protein Expression.

The pET28a vector with human p62 gene inserted was a generous gift from Maojun Yang's Lab. Subsequently, various mutants and truncations of p62 are cloned by using primers according to the following table.

Primer	Sequence, 5'-3'
P62 C331S F	CAGATGGAGTCGGATAACTCTTCAG
P62 C331S R	AGAGTTATCCGACTCCATCTGTTCC
P62 403E F	GGAAATGGGCTTCTCAGATGAAGG
P62 403E R	TGAGAAGCCCATTTCCAGCATCTG
P62 407E F	CCAGATGCTGTCAATGGGCTTC
P62 407E R	TGACAGCATCTGGGAGAGGGACTC
P62 Sortase End F	GAGAGCCGTCATTGGAAATTTCTGTAACTCG
P62 Sortase End R	CAACTCGAGTTAACCGGTTTCCGGGAGATGTGGGTACAAGG

Table S2 Primers used for cloning.

All proteins except for ubiquitin were expressed from peT28a vectors with a PreSission Protease cleavage site inserted between the N-terminal His-tag and protein gene. Whereas ubiquitin was expressed from peT22b vectors. All proteins were expressed in *E. coli* BL21 (DE3) cells grown at 37℃ until OD600 reached to 0.6, then the cells were induced with 200 mM IPTG at 18℃ for 14 hours. The cells were harvested by centrifugation at 3500 g for 30 min and then lysed mechanically on ice by sonication with 30 mL/L lysis buffer (20 mM Tris pH7.5, 150 mM NaCl). After centrifugation at 15000 g for 1.5 hours at 4℃, supernatant was incubated with 2 mL Ni Sepharose beads (GE Healthcare) for 0.5 hour. After washed by Wash Buffer (20 mM Tris pH7.5, 500 mM NaCl, 20 mM Imidazole) for 20 column volume, the desired protein was eluted from the Nickel resin by 5 column volume of Elute Buffer (20 mM Tris pH7.5, 150 mM NaCl, 250 mM Imidazole). Concentrated proteins were further purified by gel filtration (Superdex 75).

The expression and purification of K63 DiUb was based on previous reports.[1]

C. General protocol for protein hydrazide ligation.

The peptide hydrazine was dissolved in the Ligation Buffer (100 mM NaH_2PO_4 pH 2.5, 6 M Gn-HCl) for a final concentration of 1 mM. After precooled and stirred in an ice-salt bath (-10℃), 10 eq of $NaNO_2$ was added to the reaction buffer for 30 min, then 40 eq of MPAA was added to the reaction mixture immediately to convert the peptide acyl azide into peptide thioester. Finally, 1.1eq of N-terminal cysteine peptide was added to the reaction mixture, and the pH was adjusted to 6.3. The reaction mixture was stirred overnight in room temperature to complete the ligation. The reaction process was monitored and the product was further purified by RP-HPLC.[2]

D. General protocol for Sortase A mediated peptide hydrazine expression.

The expressed segment 4 contains Sortase A cleavage site (LPETG) was purified as above methods. Then 1 eq of N terminal protein and 1 eq of Sortase A was added to the Converting Buffer (20 mM Tris pH 8, 150 mM NaCl, 10 mM $CaCl_2$ and 200 mM NH_2NH_2) with a final concentration of 0.1 mM for segment 5. The reaction was stirred gently in 37℃ for 8 hours, the reaction process and the reaction product were monitored and further purified by RP-HPLC.[3]

E. Protein folding and purification.

Synthesized proteins were lyophilized and then dissolved in a small amount of Folding Buffer (6 M Gn-HCl, 100 mM Na_2HPO_4, pH 7.4) to a final concentration of 2 mg/mL. The protein solution was transferred to a dialysis bag and dialyzed with Dialysis Buffer (20 mM Tris, 150 mM NaCl, pH 7.5) until the Gn-HCl was removed from protein solution. The dialyzed protein was then concentrated by using Amicon Ultra-15 3 kDa MWCO centrifugal device (Millipore) and further purified by gel filtration (Superdex 75).

F. Circular diagram determination

CD spectra were recorded at 298 K on a Pistar π-180 CD spectrometer with a wavelength from 260 nm to 195 nm, and the concentrations of different p62 proteins were diluted to 0.1-0.2 mg/mL. Each sample was measured at least three times.

G. Surface plasmon resonance determination.

The binding force between all kinds of p62 and K63 DiUb were measured by Biacore T200 (GE Healthcare, Sweden) at 25℃. Synthetic phosphorylated p62 and expressed p62 as ligands were diluted to 0.03 mg/mL with ACE buffer, pH 4.5. Then ligand was covalently immobilized to a fresh CM5 sensor chip (GE Healthcare) immediately. The immobilization step was accomplished manually with a final response unit (RU) value of 400. The binding force

measurement was run at 30 uL/min in HBS-EP^{+} buffer (GE Healthcare). To measure the binding affinity, K63 DiUb as analyte was diluted to at least 10 concentrations with HBS-EP^{+} buffer using a double dilution method, and the concentration range was properly optimized for different substrates. The regeneration step was not needed. A variety of non-zero concentrations and a zero-concentration analyte were injected into the chip at a flow rate of 30 uL/min, and the contact and dissociation time were 60 seconds and 30 seconds respectively. The RU value was collected and all the experimental data was comprehensively analyzed by Evaluation Software (Biacore T200). Each sample was measured three times.

H. MS/MS identification of specific phosphorylation site.

For LC-MS/MS analysis of different site phosphorylated peptides, the peptide fragments were isolated by eluting with a 60 min gradient elution with a flow rate of 0.25 μL/min using a Thermo-Dionex Ultimate 3000 HPLC system, which was directly linked to the Thermo LTQ-Orbitrap Velos pro mass spectrometer. The MS/MS spectra was searched against p62 database in Proteome Discoverer (Vesion 1.4) searching algorithm.

3. Experimental figures

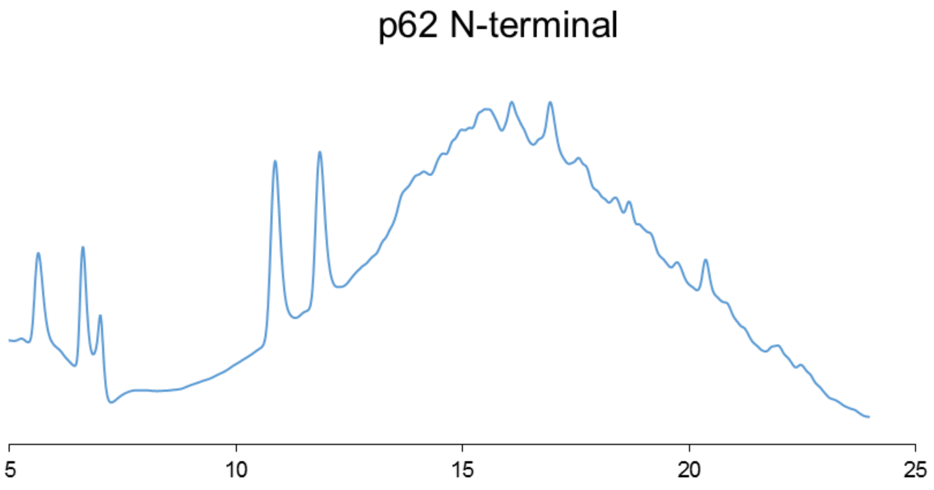

Figure S1 Analytical HPLC chromatogram of unpurified synthetic N-terminal peptide (amino acid 326-353) using standard 9-fluorenylmethoxycarbonyl-based solid phase peptide synthesis (Fmoc-SPPS).

A

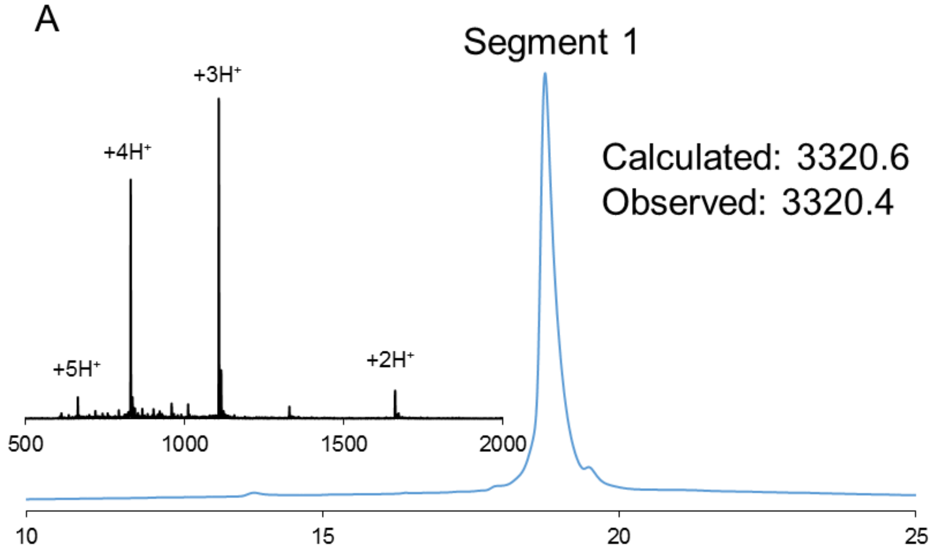
Segment 1
+3H+
+4H+
Calculated: 3320.6
Observed: 3320.4
+5H+
+2H+
500
1000
1500
2000
10
15
20
25

B

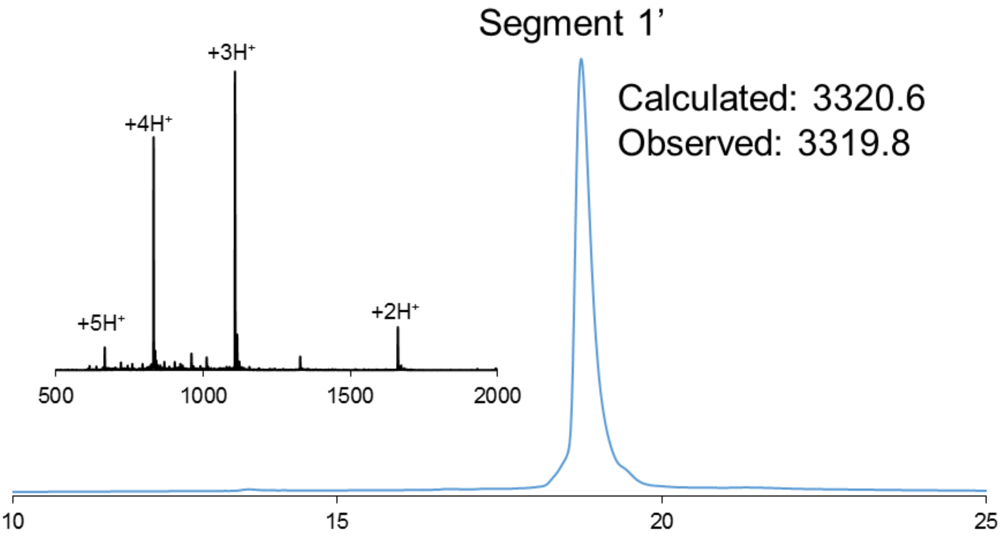
Segment 1'
+3H+
+4H+
Calculated: 3320.6
Observed: 3319.8
+5H+
+2H+
500
1000
1500
2000
10
15
20
25

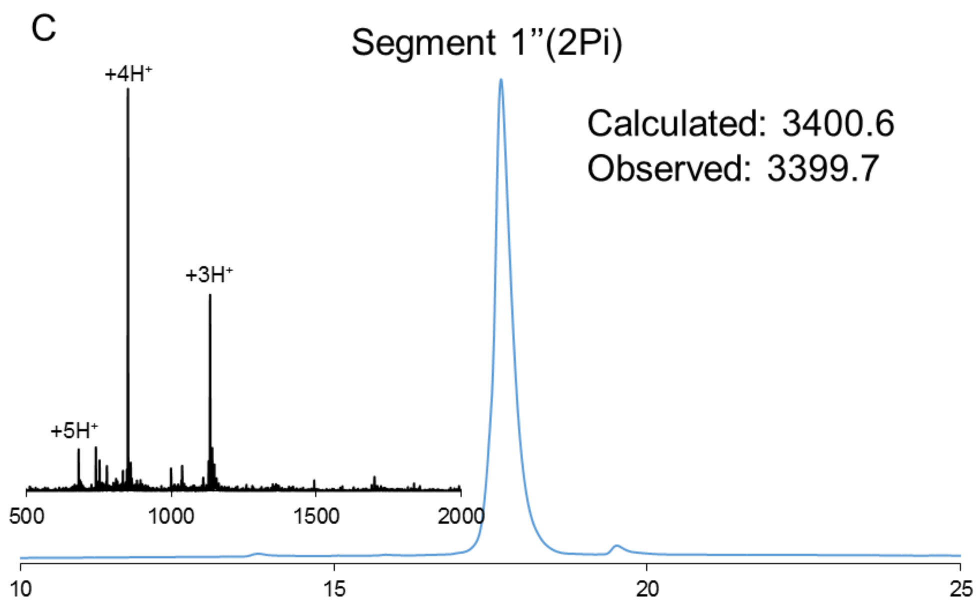

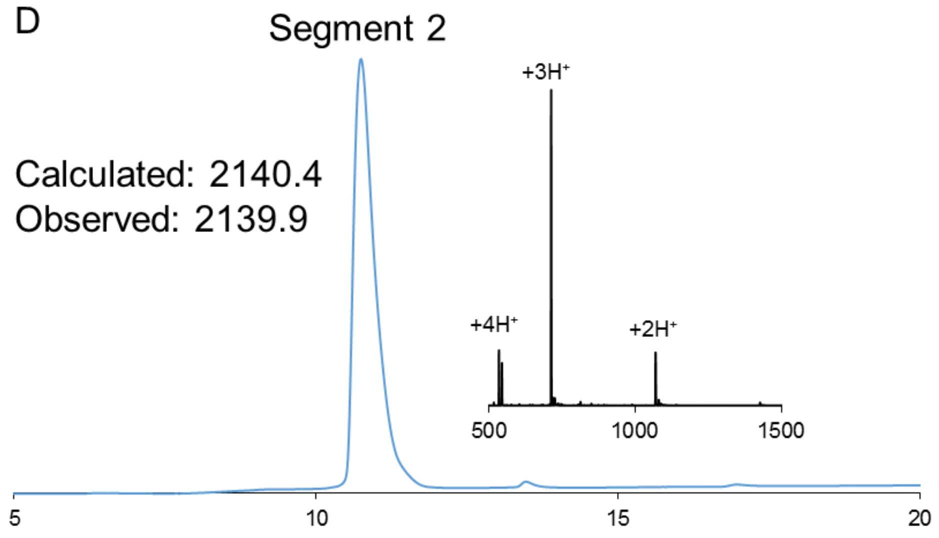

Figure S2 LC-MS characterization of synthetic peptide segments. A) Analytical HPLC chromatogram and ESI mass spectra of purified peptide Segment 1 (containing 403 phosphorylation). B) Analytical HPLC chromatogram and ESI mass spectra of purified peptide Segment 1’ (containing 407 phosphorylation). C) Analytical HPLC chromatogram and ESI mass spectra of purified peptide Segment 1” (containing both 403 and 407 phosphorylation). D) Analytical HPLC chromatogram and ESI mass spectra of purified peptide Segment 2.

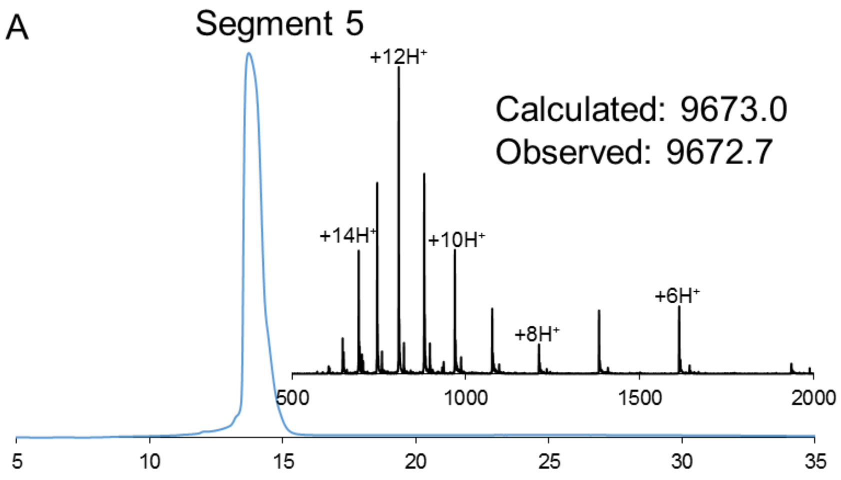

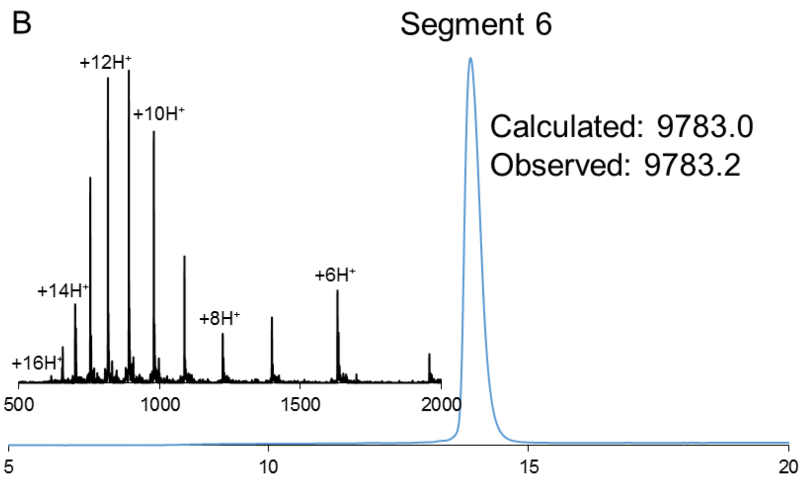

Figure S3 LC-MS characterization of Sortase A mediated peptide hydrazine expression. A) Analytical HPLC chromatogram and ESI mass spectra of purified peptide hydrazide Segment 5. B) Analytical HPLC chromatogram and ESI mass spectra of purified MesNa thioester Segment 6.

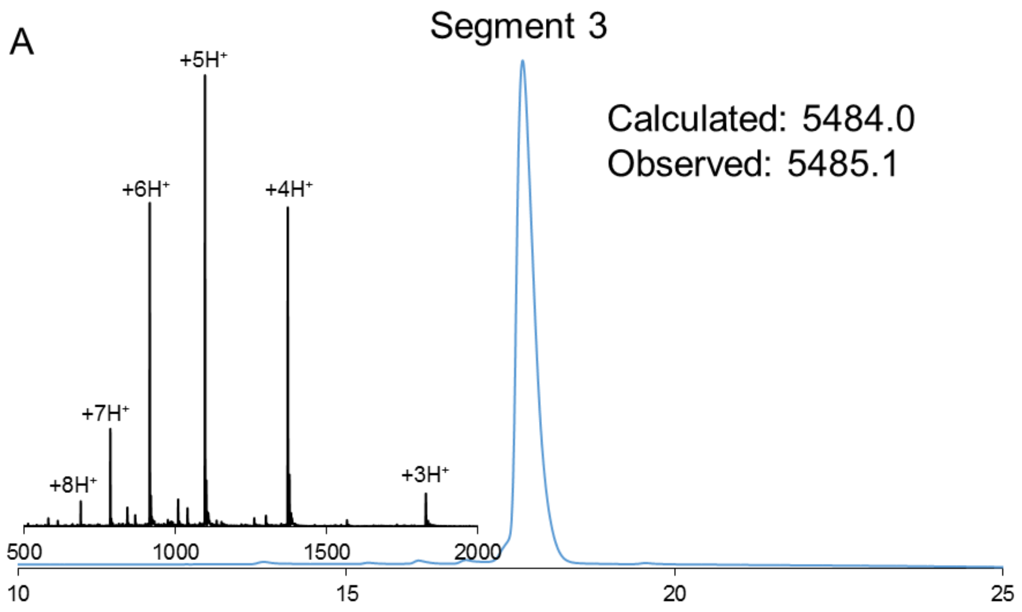
A
Segment 3
Calculated: 5484.0
Observed: 5485.1
+5H+
+6H+
+4H+
+7H+
+8H+
+3H+
500
1000
1500
2000
10
15
20
25

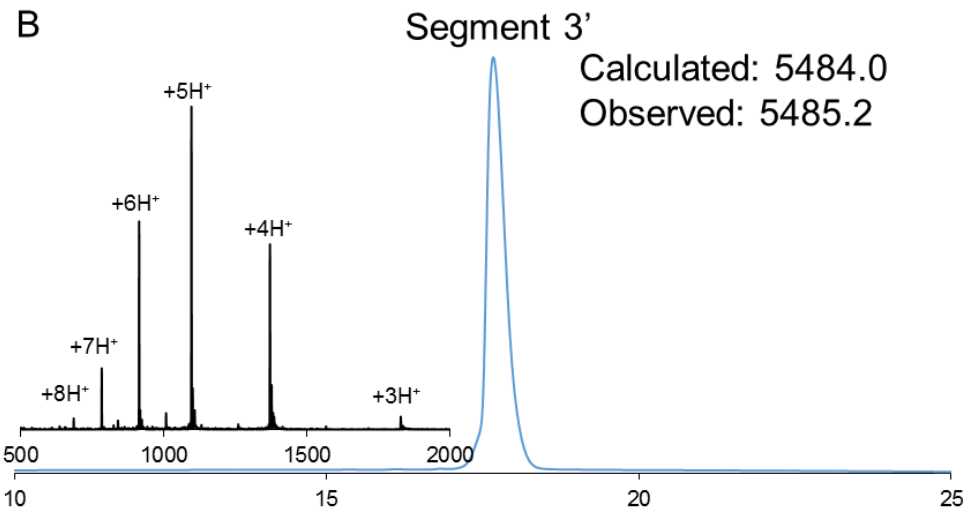
B
Segment 3'
Calculated: 5484.0
Observed: 5485.2
+5H+
+6H+
+4H+
+7H+
+8H+
+3H+
500
1000
1500
2000
10
15
20
25

C

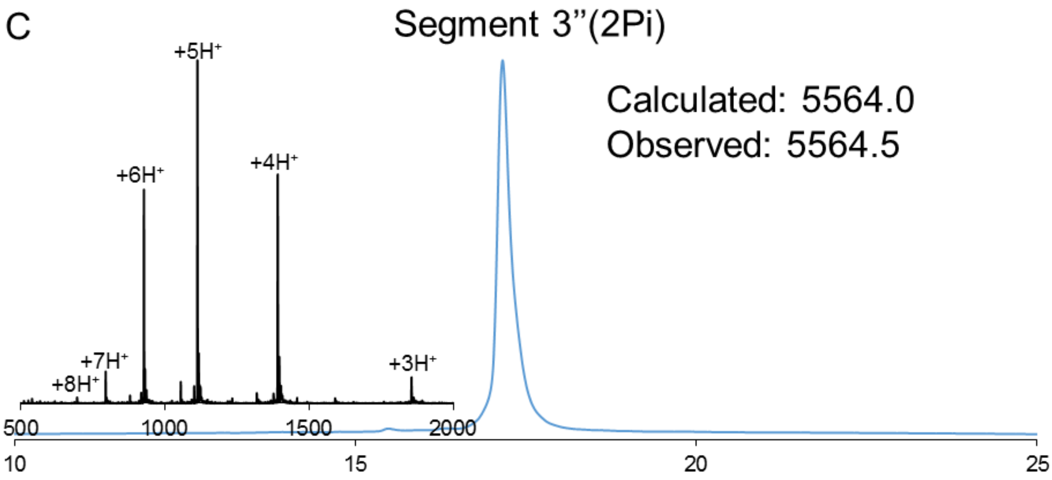
Segment 3''(2Pi)
Calculated: 5564.0
Observed: 5564.5
+5H+
+6H+
+4H+
+7H+
+8H+
+3H+
500
1000
1500
2000
10
15
20
25

D

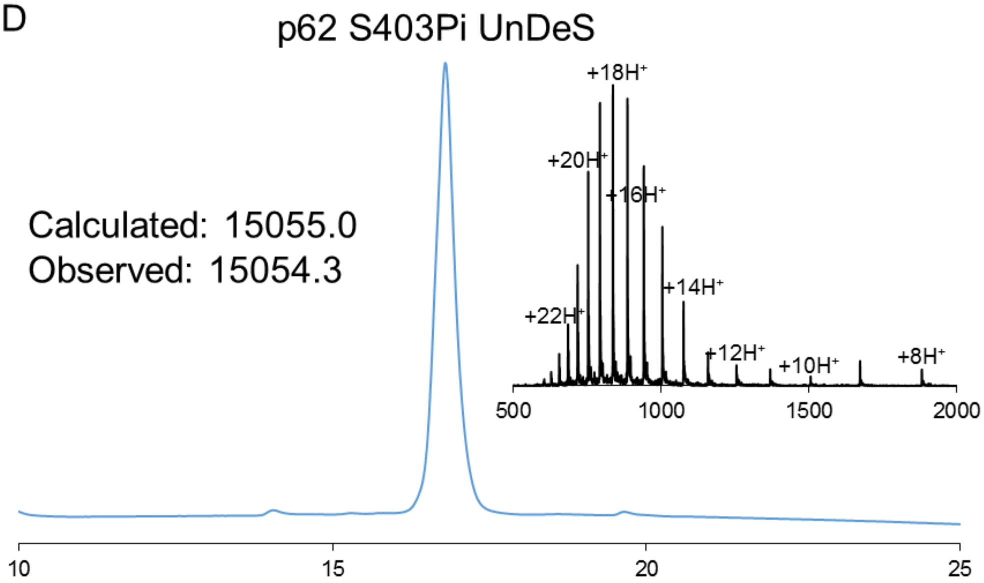
p62 S403Pi UnDeS
Calculated: 15055.0
Observed: 15054.3
+18H+
+20H+
+16H+
+14H+
+22H+
+12H+
+10H+
+8H+
500
1000
1500
2000
10
15
20
25

E

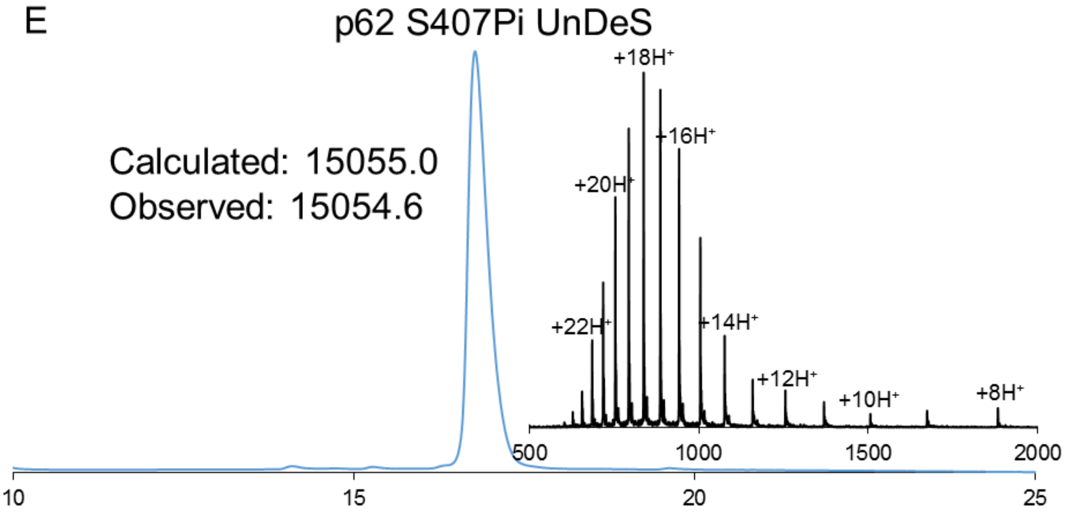

F

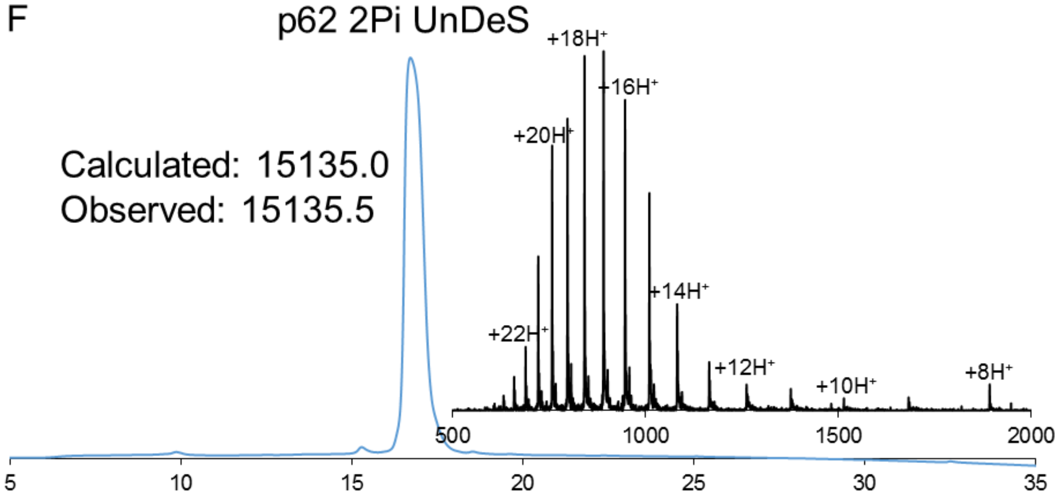

Figure S3 LC-MS characterization of protein hydrazide ligation. A) Analytical HPLC chromatogram and ESI mass spectra of purified ligation product peptide Segment 3 (containing

403 phosphorylation and Cys to Gln reverse mutation with a sulfur atom substitution). B) Analytical HPLC chromatogram and ESI mass spectra of purified ligation product peptide Segment 3' (containing 407 phosphorylation and Cys to Gln reverse mutation with a sulfur atom substitution). C) Analytical HPLC chromatogram and ESI mass spectra of purified ligation product peptide Segment 3'' (containing both 403 and 407 phosphorylation and Cys to Gln reverse mutation with a sulfur atom substitution). D) Analytical HPLC chromatogram and ESI mass spectra of purified ligation product p62 S403Pi with a Cys mutation temporarily at the ligation site. E) Analytical HPLC chromatogram and ESI mass spectra of purified ligation product p62 S407Pi with a Cys mutation temporarily at the ligation site. F) Analytical HPLC chromatogram and ESI mass spectra of purified ligation product p62 2Pi with a Cys mutation temporarily at the ligation site.

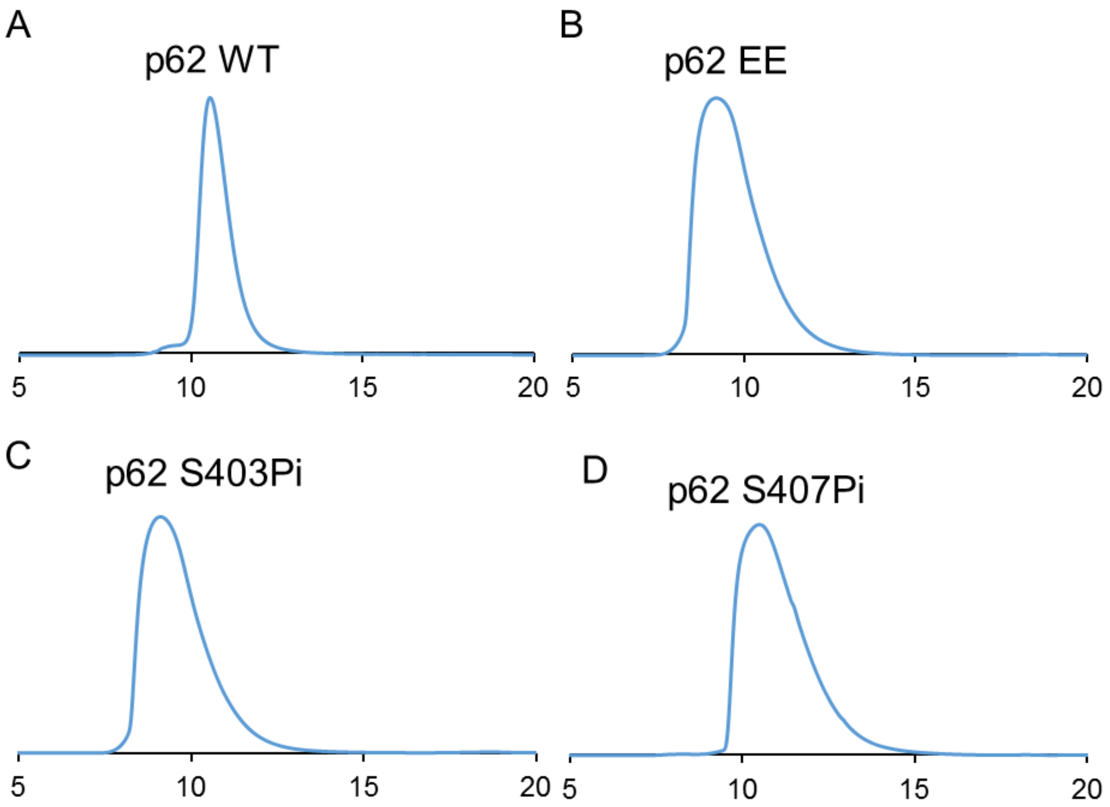

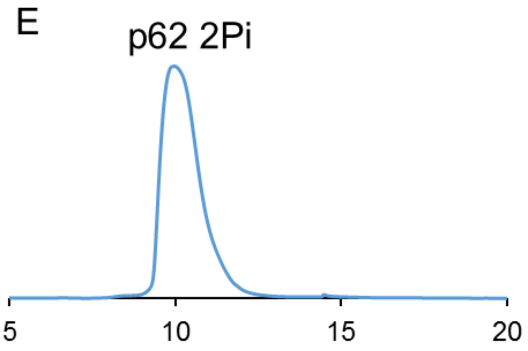

Figure S4 Gel filtration chromatogram of synthetic and expressed proteins. A) Expressed p62 WT. B) Expressed p62 EE. C) Synthetic p62 with S403 site phosphorylation. D) Synthetic p62 with S407 site phosphorylation. E) Synthetic p62 with both S403 and S407 site phosphorylation.

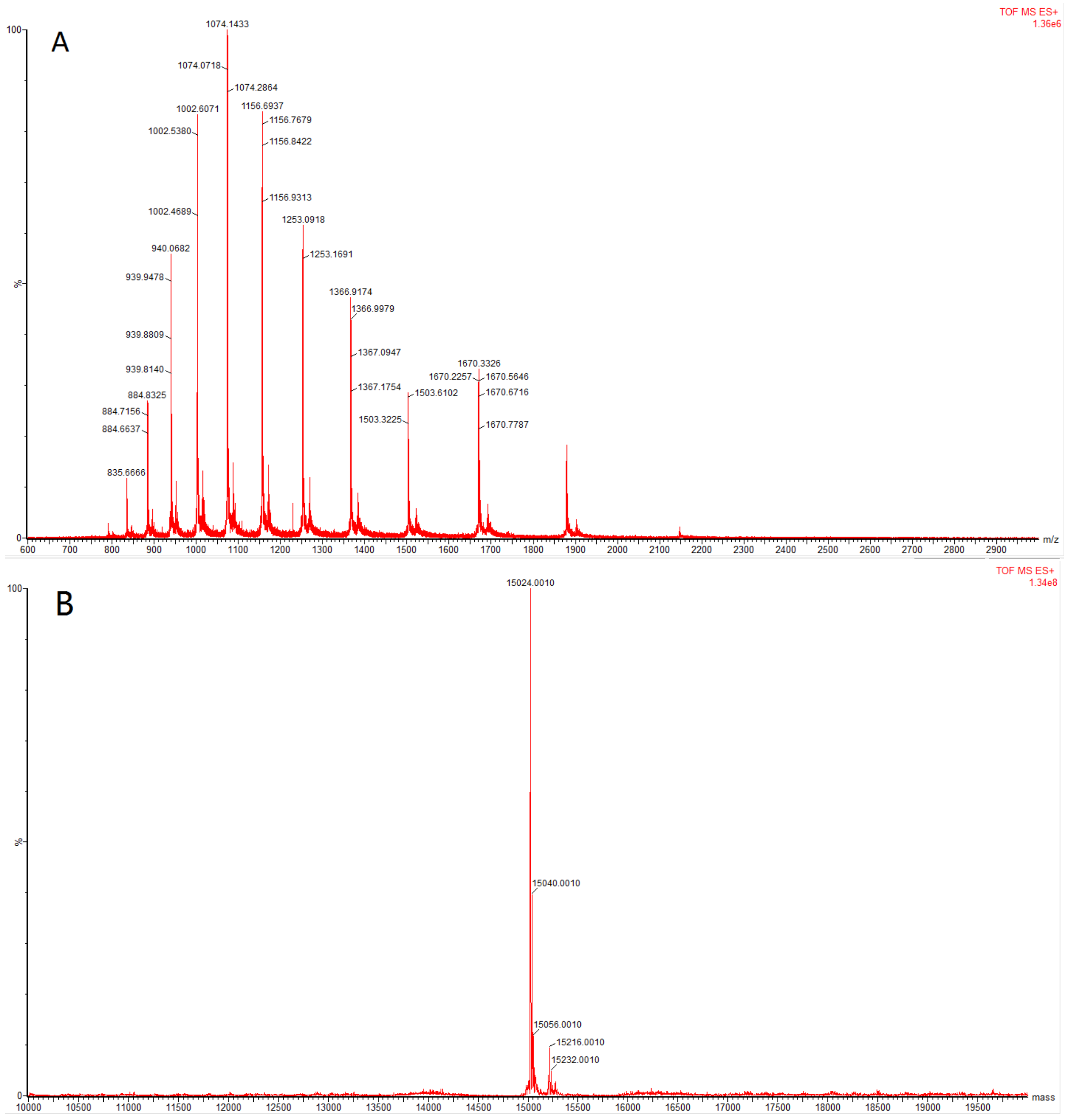

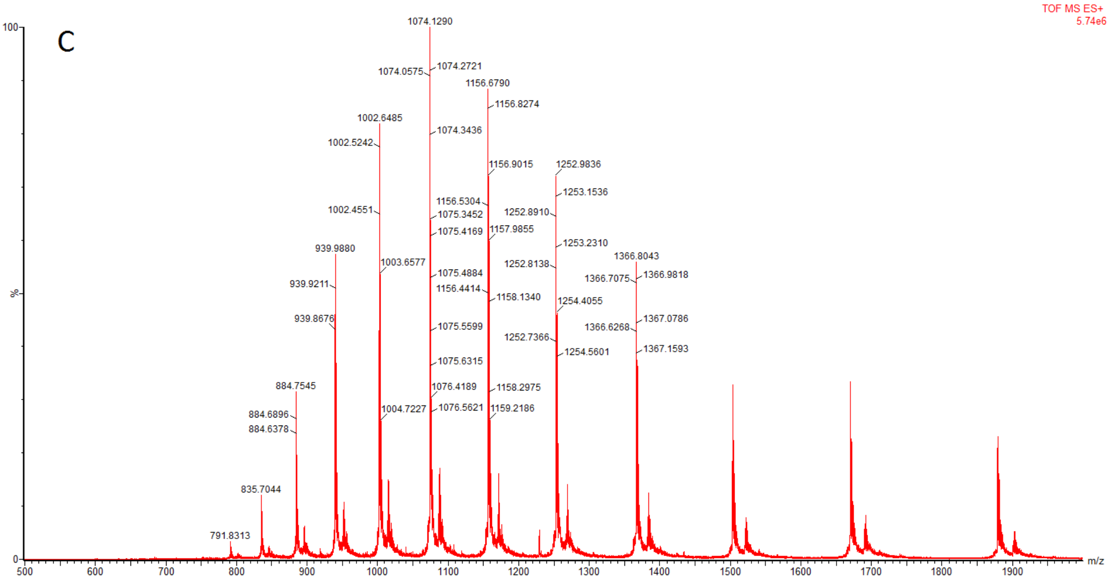
C
TOF MS ES+
5.74e6
1074.1290
1156.6790
1002.6485
1252.9836
1366.8043
939.9880
884.7545
835.7044
791.8313
m/z

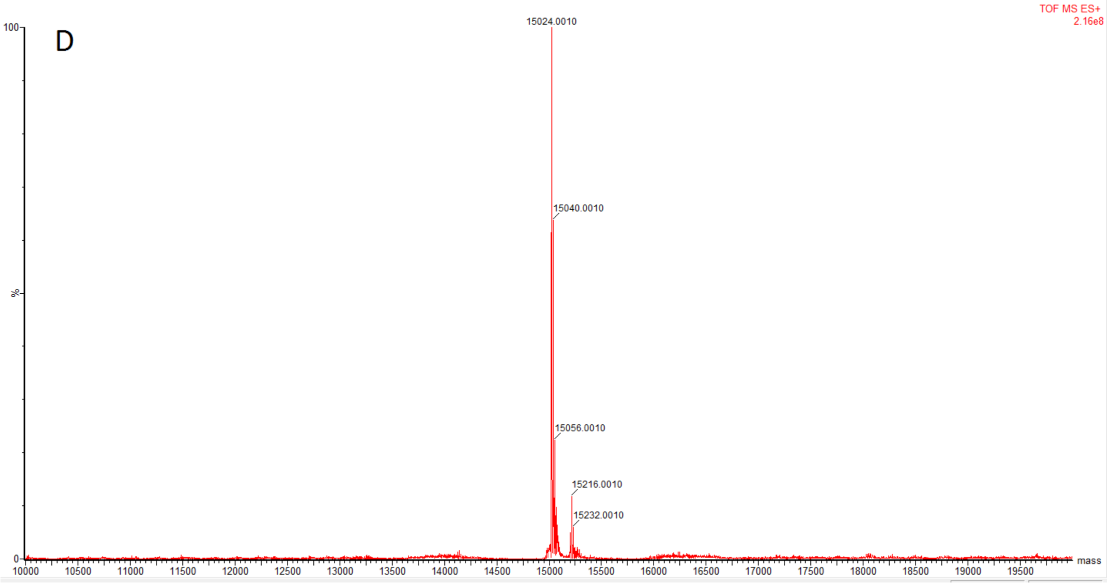
D
TOF MS ES+
2.16e8
15024.0010
15040.0010
15056.0010
15216.0010
15232.0010
mass

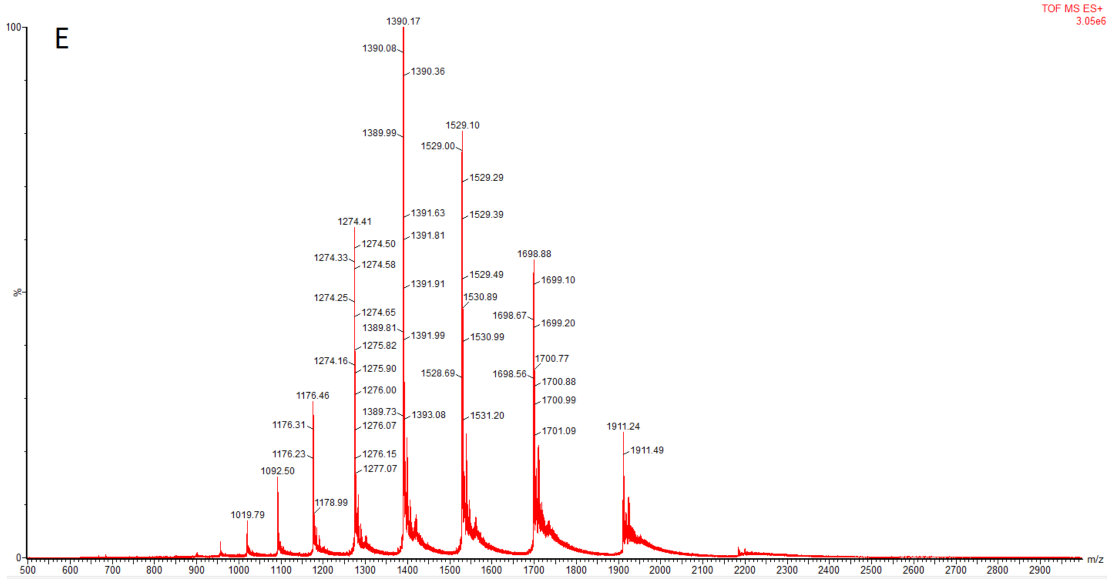
E
TOF MS ES+
3.05e6
1390.17
1529.10
1274.41
1698.88
1176.46
1911.24
1092.50
1019.79
m/z

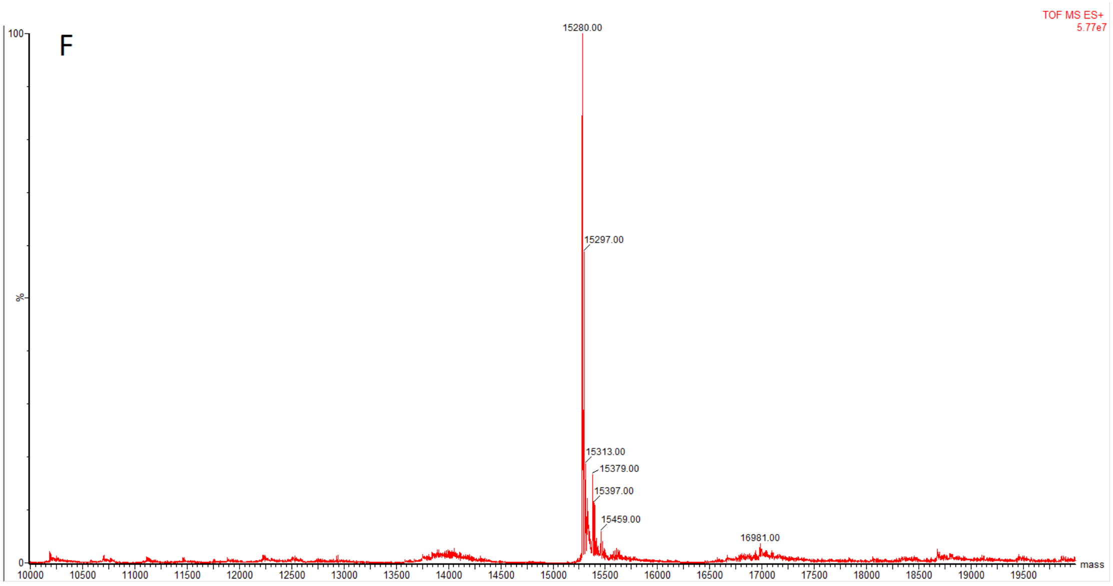

Figure S5 High resolution mass spectra of synthetic proteins. A) High resolution ESI-MS spectra of synthetic p62 S403Pi. B) Deconvolution mass spectra of synthetic p62 S403Pi. C) High resolution ESI-MS spectra of synthetic p62 S407Pi. B) Deconvolution mass spectra of synthetic p62 S407Pi. E) High resolution ESI-MS spectra of synthetic p62 2Pi. F) Deconvolution mass spectra of synthetic p62 2Pi.

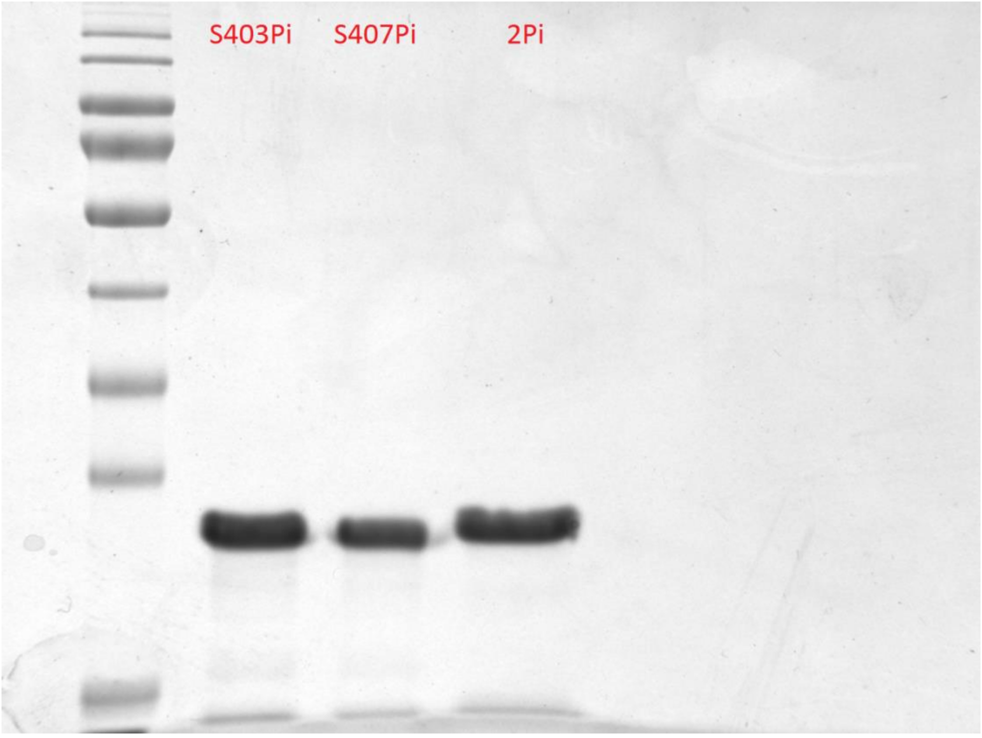

Figure S6 SDS-PAGE of synthetic p62 proteins.

Reference:

1 A. P. VanDemark, R. M. Hofmann, C. Tsui, C. M. Pickart and C. Wolberger, *Cell*, 2001, **105**, 711-720.

2 G. M. Fang, Y. M. Li, F. Shen, Y. C. Huang, J. B. Li, Y. Lin, H. K. Cui and L. Liu, *Angew Chem Int Ed Engl*, 2011, **50**, 7645-7649.

3 Y. M. Li, Y. T. Li, M. Pan, X. Q. Kong, Y. C. Huang, Z. Y. Hong and L. Liu, *Angew Chem Int Ed Engl*, 2014, **53**, 2198-2202.
